# A Novel Cerium-Loaded Amyloid Hybrid Membrane for Advanced Removal of Fluorine-18 in Medical Wastewater

**DOI:** 10.3390/toxics14060490

**Published:** 2026-06-03

**Authors:** Yue Xing, Fan Zhang, Xu Zhang, Yuezhou Wei, Chengtao Yue, Xiangbiao Yin

**Affiliations:** 1 School of Nuclear Science and Technology, University of South China, 28 Changsheng West Road, Hengyang 421001, Chinayzwei@usc.edu.cn (Y.W.); chengtaoyue@usc.edu.cn (C.Y.); 2 Division of Radiation Protection and Safety Control, Cyclotron and Radioisotope Center, Tohoku University, Sendai 980-8578, Japan; 3 School of Nuclear Science and Engineering, Shanghai Jiao Tong University, Shanghai 200240, China; 4 Key Laboratory of Advanced Nuclear Energy Design and Safety, Ministry of Education, University of South China, Hengyang 421001, China

**Keywords:** fluorine-18, adsorptive removal, amyloid fibril, cerium oxide, hybrid membrane

## Abstract

Despite its critical role in disease diagnosis as a radiopharmaceutical, Fluorine-18 generates medical wastewater that necessitates efficient treatment, for which membrane adsorption stands out as a potent method, albeit one that demands high-performance membranes with exceptional permeability and adsorption capacity. This study presents a novel cerium-loaded amyloid fibril hybrid membrane designed for efficient removal of fluorine-18 from such wastewater. The membrane is fabricated through a facile process involving oxidation–precipitation of cerium species onto amyloid fibrils, followed by vacuum filtration, with further compositional tuning via incorporation of porous silica or activated carbon dopants. The resulting membrane retains the characteristic amyloid fibril structure and exhibits high water permeability with a flux of up to 803.3 L/(m^2^·h·bar), superior to most of the other membrane materials. It effectively removes fluoride ions (F^−^) from both low and high-concentration solutions, achieving a removal efficiency of up to 99% and a maximum adsorption capacity of 580 mg/g, outperforming many existing membrane materials. The hybrid membrane also demonstrates notable resistance to ionic interference, enabling selective F^−^ adsorption from solutions containing high concentrations of Cl^−^, NO_3_^−^ and SO_4_^2−^, with a distribution coefficient (Kd) as high as 4.1 × 10^4^ mL/g; furthermore, it maintains a fluoride removal rate above 51% after ten consecutive adsorption cycles. The membrane retains 51% of its initial fluoride removal efficiency after 10 cycles, indicating potential for repeated use, although further optimization or regeneration strategies would be required to fully restore performance. Mechanistic investigations reveal that F^−^ adsorption occurs mainly through ion exchange with hydroxyl groups on CeO_2_. This work introduces a promising novel material with significant potential for the efficient treatment of medical radioactive wastewater containing fluorine-18.

## 1. Introduction

F-18 medical wastewater primarily originates from the nuclear medicine department of hospitals during the production and use of fluorine-18 labeled radiopharmaceuticals (such as FDG) [1,2,3,4,5,6,7], including liquid waste generated from cleaning equipment and containers, as well as from flushing patient excreta [8]. Typical cyclotron production yields of FDG can exceed tens of GBq per batch, depending on the specific cyclotron parameters and irradiation conditions, with daily multi-patient clinical use producing several GBq. The core hazard lies in the fluorine-18 isotope present in the water, which emits positrons and gamma rays. Although its half-life is relatively short, approximately 110 min, and it decays relatively quickly, it still possesses significant radioactivity before complete decay [9,10,11,12]. If improperly discharged into the environment without adequate treatment, it could not only cause radiological impacts on aquatic ecosystems but also potentially threaten public health through environmental cycles [13,14,15]. The most common current treatment method is “decay storage,” which leverages the short half-life characteristic by collecting the wastewater in specially designed shielded decay tanks. The wastewater is stored for a sufficiently long time (typically over 10 half-lives, about 20 h) until its radioactivity level naturally decays to below the nationally mandated safety exemption limit before discharge [16]. However, the main drawbacks of this method include high demands for storage space and turnover efficiency, especially for large medical centers that generate substantial daily wastewater volumes, necessitating the construction of multiple large, well-shielded decay tanks for rotational use. The initial construction and maintenance costs are high, and the method is inherently passive, unable to cope with unexpectedly large wastewater volumes or accelerated treatment needs. Additionally, strict monitoring and management during storage are required to prevent leakage risks [16,17,18]. Therefore, actively researching and developing more efficient, compact, and responsive active treatment technologies is of great significance for improving the operational efficiency of medical institutions, reducing environmental risks, and ensuring public health safety.

Current treatment methods for fluoride-containing wastewater mainly include chemical precipitation [19], adsorption [20,21], ion exchange [22], and membrane separation [23]. Among these, membrane separation has garnered widespread attention due to its high efficiency [24]. The core principle of membrane separation lies in the selective separation mechanism of specific functional membrane materials. Through active adsorbents (such as metal-organic frameworks, nano-ceria, etc.) loaded on the membrane surface or within the membrane pores, specific chemical adsorption or ion exchange occurs with fluoride ions, enabling efficient capture and enrichment of fluoride ions, thereby achieving deep purification. When applying membrane adsorption technology to F-18 medical wastewater treatment, its significant advantage is the ability to rapidly retain the fluoride ions on the membrane surface or within the pores, enabling continuous and rapid purification of F-18 medical wastewater. This overcomes the drawbacks of traditional decay tanks, such as long storage cycles and large space requirements, making it particularly suitable for medical institutions with limited space. The core driving force of membrane adsorption separation technology is the design of high-efficiency membrane materials, whose performance depends on three key indicators: high permeability to ensure treatment flux, high selectivity to achieve precise recognition of fluoride ions and high adsorption capacity to ensure long-term stable operation. Currently, membrane materials developed for fluoride ion adsorption mainly include modified polymer membranes, inorganic-organic hybrid membranes and novel nanocomposite membranes [24,25]. However, the practical application of these materials in F-18 wastewater treatment faces challenges related to selectivity, permeability and adsorption capacity, where competing ions can impair targeting efficiency, fouling often reduces treatment flux, and limited adsorption sites lead to rapid saturation necessitating frequent regeneration, thereby compromising long-term operational stability. Developing new membrane materials that synergistically enhance selectivity, permeability, and adsorption capacity is key to overcoming the current technical bottlenecks.

Amyloid fibril hybrid membranes demonstrate significant advantages in the field of separation materials due to their excellent membrane-forming ability and functional integration capabilities [26,27]. These membranes utilize self-assembled amyloid fibrils as a structural framework, forming a three-dimensional network with high specific surface area and interconnected channels through processes such as interfacial assembly, vacuum filtration, or biomimetic mineralization. Their hierarchical porous structure and tunable surface chemical properties provide an ideal platform for functional modifications. By incorporating functional components such as inorganic nanoparticles, polymers, or carbon materials, the separation performance of the membranes can be effectively enhanced, for instance, by increasing porosity or introducing specific recognition sites. Notably, the co-integration of porous materials (e.g., silica, activated carbon) and ion-recognition active components into the amyloid fibril network enables further optimization of membrane performance [28]. This multi-scale regulation strategy not only preserves high permeability but also grants precise recognition capability for target ions, significantly improving separation efficiency and selectivity. Based on this approach, the directional incorporation of porous fillers and the introduction of fluoride-specific adsorption sites allow for the preparation of amyloid fibril hybrid membranes with both high permeability and selectivity, offering an efficient and reliable technical solution for the treatment of F-18 medical wastewater.

This research focused on the design and synthesis of a high-performance amyloid fibril hybrid membrane for the targeted removal of fluorine-18 from medical wastewater. The membrane was fabricated using beta-lactoglobulin-based amyloid fibrils formed through vacuum filtration, with cerium oxide, which is known for its strong affinity toward fluoride ions, loaded via in situ oxidative precipitation. To improve permeability, hydrophilic silica particles (35–70 μm) and activated carbon were incorporated as fillers during the synthesis process to increase porosity during the vacuum filtration of amyloid fibers for membrane formation, thereby preventing dense packing and the resulting low porosity, while their hydrophilicity also contributed to enhancing the overall membrane hydrophilicity. The structural, morphological, and compositional characteristics of the resulting hybrid membrane were systematically characterized using X-ray diffraction (XRD), atomic force microscopy (AFM), transmission electron microscopy (TEM) and Fourier-transform infrared spectroscopy (FT-IR). Water flux measurements were conducted to evaluate its permeability, while batch adsorption experiments assessed its fluoride ion removal efficiency from aqueous solutions. Finally, X-ray photoelectron spectroscopy analysis was employed to elucidate the underlying adsorption mechanism of fluoride ions by the membrane.

## 2. Materials and Methods

### 2.1. Materials

Porous silica was supplied by Hengyang Chemical Co., Ltd. (Hengyang, China) and had a particle diameter range of 36–75 μm. Cerium chloride heptahydrate (CeCl_3_·7H_2_O, AR, ≥99%) was obtained from Shanghai Macklin Biochemical (Shanghai, China). β-Lactoglobulin (BLG, purity > 95%) and the anion exchange resin IRA-900 were purchased from Shanghai Aladdin Bio-chem Technology (Shanghai, China). The standard solution of 18F was provided by China Isotope & Radiation (Beijing, China). Commercially available activated carbon was supplied by Zhongliao Environmental Protection Technology (Zhengzhou, China). Hydrochloric acid (GR, 36.0~38.0%) was purchased from National Pharmaceutical Group Chemical Reagents (Shanghai, China). Hydrogen peroxide (AR, ≥30%) was purchased from National Pharmaceutical Group Chemical Reagents (Shanghai, China). All chemicals were used as received without further purification.

### 2.2. Preparation of Cerium-Loaded Amyloid Fiber Hybrid Membrane

The β-lactoglobulin amyloid fibrils (LAFs) were synthesized based on established protocols with modifications. Initially, commercial β-lactoglobulin powder was purified to obtain the monomeric form. Briefly, a 10 wt% protein solution was prepared in ultrapure water and adjusted to pH 4.6, followed by extensive dialysis (MWCO: 8 kDa) against ultrapure water for 5–7 days to remove low-molecular-weight impurities. After dialysis, the sample was freeze-dried, and the purified protein was stored at 4 °C as the starting material for fibril formation. Subsequently, 2 g of the purified β-lactoglobulin monomer was dissolved in ultrapure water to form a 2 wt% protein solution. The pH was adjusted to 2 using 1 mol/L HCl, and the solution was heated at 90 °C for 5 h to form β-lactoglobulin amyloid fibrils (LAFs) [27,28,29].

**Figure 1:**
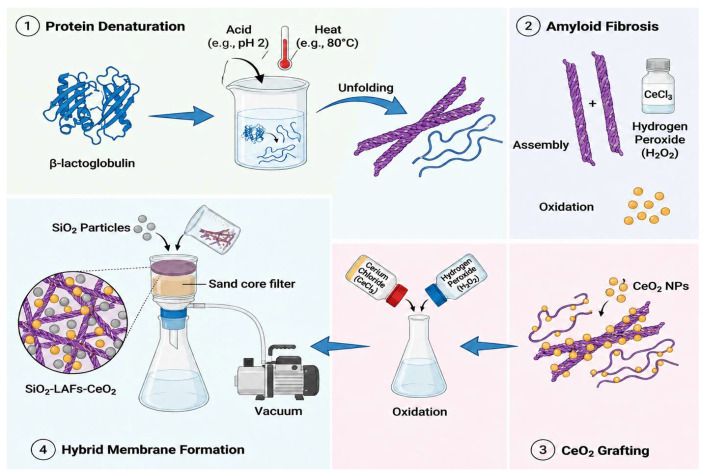
Synthetic procedures illustration for the amyloid fibril hybrid membrane.

For the synthesis of the cerium-loaded amyloid fiber hybrid membrane, a certain amount of cerium chloride (7–30 mmol) was added to 10 mL of the LAFs suspension (2 wt%) and stirred at room temperature for 2 h. Then, 10 mL of H_2_O_2_ solution was introduced, and stirring continued for another hour, yielding a cerium-loaded amyloid fiber solution (Figure 1). The resulting mixture was vacuum-filtered to form a CeO_2_-loaded amyloid fiber hybrid membrane (LAFs-CeO_2_). Additionally, LAFs-CeO_2_ membranes doped with either large-pore silica or activated carbon were also prepared. Typically, 0.08 g of sieved SiO_2_ was mixed with 2 mL of the LAFs-CeO_2_ solution. After stirring at room temperature for 1 h, the mixture was vacuum-filtered to produce a SiO_2_-doped LAFs-CeO_2_ membrane (SiO_2_-LAFs-CeO_2_). Similarly, an activated carbon-doped LAFs-CeO_2_ membrane (AC-LAFs-CeO_2_) was synthesized using an analogous procedure.

### 2.3. Analysis and Characterization

The morphology of the amyloid fibrils (LAFs) was characterized using atomic force microscopy (AFM; Bruker, Leiderdorp, The Netherlands) and transmission electron microscopy (TEM; JEOL JEM-2100Plus, Tokyo, Japan). The composition and chemical state of the LAFs were analyzed by X-ray photoelectron spectroscopy (XPS, ESCALAB Xi+, Waltham, MA, USA) with monochromatic Al Kα radiation at 200 W. The resulting data were calibrated and fitted using XPS-peak software. The chemical structure of the LAFs was identified by Fourier-transform infrared spectroscopy (FT-IR; Nexus 870, Madison, WI, USA). The thermal stability of the LAFs was assessed via thermogravimetric analysis (TGA; NETZSCH STA 449 F3, Bayern, Germany). Additionally, powder X-ray diffraction (XRD; Rigaku D/max-2550pc, Tokyo, Japan) patterns were acquired over a 2θ range of 0° to 90°.

### 2.4. Water Flux Measurement

For testing, the membrane is carefully mounted in a filtration cell, either freestanding on a porous support or already supported on a substrate, ensuring a tight seal to prevent leaks. The system is then primed by flushing with deionized water, followed by a pre-compaction phase at a higher pressure to stabilize the membrane structure and ensure reproducible results. Once stabilized, the transmembrane pressure is set to a constant value (1 bar), and the filtration time is measured by collecting the permeate volume over a fixed period or timing the collection of a specific volume, with multiple repetitions for accuracy. After data collection, the system is depressurized gradually to avoid membrane damage, and the key parameters, including volume collected, membrane area, time, and pressure, are used to calculate the water flux (*Jw*) were calculated using Equation (1) and subsequently the normalized water permeability (*NWP*) were calculated using Equation (2) for comparative analysis [30].

(1) $$Jw=\frac{V}{A\times t}$$

(2) $$NWP=\frac{Jw}{∆P}$$

where *V* (L) corresponds to the volume of permeate water collected, *A* (m^2^) denotes the effective membrane area, *t* (h) specifies the filtration time, *Jw* (L/(m^2^·h)) represents the water flux, Δ*P* (bar) is the applied trans-membrane pressure difference, and *NWP* (L/(m^2^·h·bar)) denotes the normalized water permeability.

### 2.5. Batch Filtration Experiment

In this study, the fluorine-18 removal efficiency of amyloid fibril-based hybrid membranes was evaluated using the bottle-point filtration technique with fluoride ions (F^−^) as a non-radioactive model. The assessment began by measuring the water flux of membranes prepared with varying fibril contents, in both freestanding form and supported on different substrates, through corresponding filtration time measurements. Subsequently, the F^−^ removal performance of different hybrid materials fabricated on the same substrate was compared. This involved filtering solutions with identical fluoride concentration and volume, followed by determining the residual F^−^ concentrations before and after filtration using ion chromatography. Furthermore, a comprehensive evaluation of the hybrid membrane’s adsorption capability was carried out, covering pH dependence, saturated adsorption capacity, reusability, selectivity and performance in fluoride-containing water samples. The corresponding saturated adsorption capacity (*Q_e_*, mg/g) and removal rate (*R*, %) were calculated using the following Equations (3) and (4) [31]:

(3) $$Q_{e}=\frac{C_{0}-C_{e}}{m}\times V$$

(4) $$R=\frac{C_{0}-C_{e}}{C_{0}}\times 100\%$$

where *C*_0_ (mg/L), *C_e_* (mg/L) are the F-concentration before the filtration and after filtration, respectively; *m* (g) is the mass of the fibrils of the hybrid membrane; *V* (L) is the volume of filtered solution.

## 3. Results and Discussion

### 3.1. Characterization

**Figure 2:**
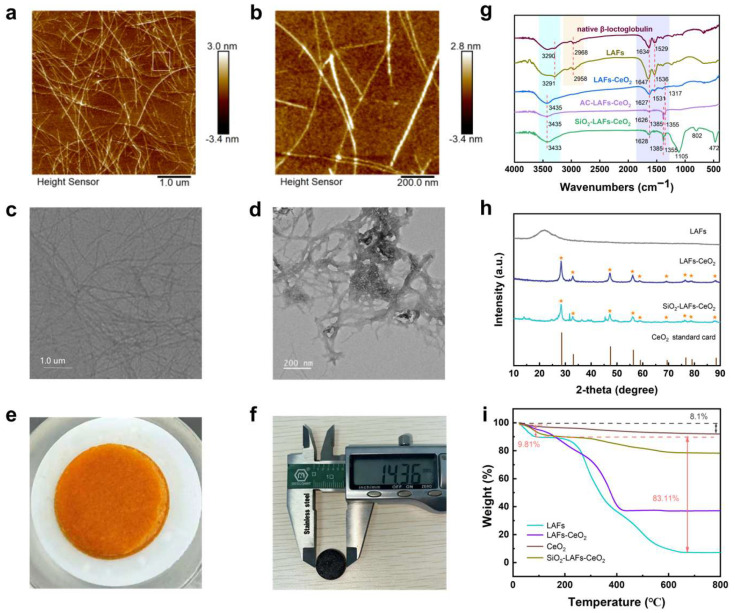
Characterization of hybrid membranes. (**a**,**b**) AFM images of LAFs. (**c**) TEM images of LAFs. (**d**) TEM images of LAFs-CeO_2_. (**e**) Photograph of SiO_2_-LAFs-CeO_2_. (**f**) Photograph of AC-LAFs-CeO_2_. (**g**) FT-IR spectra of different hybrid membranes. (**h**) XRD patterns of different hybrid membranes. (**i**) TG analysis of different hybrid membranes.

The morphology of both LAFs and LAFs-CeO_2_ was characterized using atomic force microscopy (AFM) and transmission electron microscopy (TEM). As shown in Figure 2a, the LAFs exhibit a well-defined fibrillar structure and are homogeneously dispersed. The fibrils possess lengths on the micrometer scale and display consistent diameters. A higher magnification image (Figure 2b) reveals that the individual fibrils have uniform diameters ranging from 20 to 30 nm and lengths extending to the micrometer scale, forming a highly interconnected three-dimensional network. This network has high porosity, conducive to enhanced water permeability. TEM observations further confirm the uniform, well-dispersed fibrous nature of the LAFs (Figure 2c). Upon loading F^−^-affinity CeO_2_, the fibril diameters increase to 50–70 nm and partial agglomeration occurs, indicating successful deposition of CeO_2_ onto the LAFs (Figure 2d). The increase in diameter and surface roughness suggests that CeO_2_ is effectively anchored, which enhances the number and accessibility of active sites for fluoride adsorption. For the doped membranes (SiO_2_-LAFs-CeO_2_ and AC-LAFs-CeO_2_), AFM and TEM show a more heterogeneous morphology with well-dispersed dopants creating additional mesoporous structures. These features facilitate water transport, increase permeability, and maintain F^−^ adsorption sites, thus supporting both high flux and superior fluoride removal efficiency. Figure 2e,f presents the morphologies of silica and activated carbon-doped LAFs-CeO_2_ hybrid membranes, respectively. The SiO_2_-LAFs-CeO_2_ membrane appears as an opaque orange-yellow film composed of loosely packed rough particles with a certain thickness. In contrast, the AC-LAFs-CeO_2_ membrane is an opaque black film. The coloration of both hybrid membranes is significantly influenced by their respective dopants.

The structures of LAFs, LAFs-CeO_2_ and SiO_2_-LAFs-CeO_2_ were analyzed using FT-IR, respectively. The FT-IR spectrum of LAFs shows strong absorption peaks at 3435 and 3290 cm^−1^, attributed to the overlapping stretching vibrations of the -OH and -NH groups from the amino acid residues of β-lactoglobulin (Figure 2g). Additionally, a stretching vibration peak of alkyl C-H bonds is observed at 2958 cm^−1^ [32], while the absorption peaks at 1647 cm^−1^ and 1536 cm^−1^ correspond to the stretching vibration of amide C=O bonds and the in-plane bending vibration of N-H bonds [33], respectively. This demonstrates the successful loading of CeO_2_ onto the LAFs’ surface and indicates a strong electronic interaction between CeO_2_ and the surface amide bonds. This result confirms that LAFs can achieve efficient loading of CeO_2_ through electrostatic interaction. After loading with CeO_2_, the FT-IR spectrum of LAFs-CeO_2_ exhibits a characteristic absorption peak at 1317 cm^−1^, assigned to the Ce-O bond stretching vibration of CeO_2_. Moreover, the absorption peak of the C=O stretching vibration, originally at 1647 cm^−1^, undergoes a red shift to 1627 cm^−1^. This demonstrates the successful loading of CeO_2_ onto the LAFs’ surface and indicates a strong electronic interaction between CeO_2_ and the surface amide bonds, demonstrating that CeO_2_ is effectively anchored and provides active sites for fluoride adsorption. After doping with AC and SiO_2_, the FTIR spectra showed a significant reduction in the characteristic absorption peaks of LAFs and CeO_2_, accompanied by the emergence of distinctive peaks corresponding to the dopants. Specifically, the AC-LAFs-CeO_2_ hybrid exhibited a strong absorption peak at 1385 cm^−1^, attributed to C-H bending vibrations or aromatic skeletal vibrations in AC. In contrast, the SiO_2_-LAFs-CeO_2_ sample displayed a more intense peak at 1105 cm^−1^, ascribed to the stretching vibrations of Si-O bonds, indicating successful incorporation of SiO_2_ [34]. FT-IR results confirm that the LAFs are loaded with a porosity-regulating component (SiO_2_) and a F^−^-affinity functional component (CeO_2_), which is critical for achieving high-efficiency fluoride adsorption.

The XRD patterns of LAFs-CeO_2_ exhibit well-defined diffraction peaks at 2θ values of approximately 28.5°, 33.1°, 47.5°, and 56.3°, which correspond to the (111), (200), (220), and (311) planes of CeO_2_ [35], respectively (Figure 2h). The absence of any extraneous peaks indicates high phase purity, confirming that CeO_2_ crystallites formed uniformly without detectable impurities or secondary phases. Such high phase purity is essential for adsorption performance, as uniform CeO_2_ crystallites ensure a consistent distribution of surface hydroxyl groups (-OH), which serve as active sites for ion-exchange interactions with fluoride ions, thereby enabling predictable and reproducible adsorption behavior. Moreover, the relatively narrow peak widths suggest a larger crystallite size, which enhances chemical stability and minimizes the risk of CeO_2_ leaching during repeated filtration cycles. In the SiO_2_-LAFs-CeO_2_ hybrid membranes, the appearance of additional diffraction peaks attributable to crystalline silica confirms the successful incorporation of the dopant without disrupting the CeO_2_ lattice. This structural integration preserves active adsorption sites while simultaneously improving membrane porosity, contributing to enhanced water flux and overall performance. Collectively, the XRD results not only verify the successful synthesis of the target hybrid membrane but also provide a robust structural basis for its high fluoride removal efficiency, selectivity, and long-term operational stability. TG analysis was then conducted to investigate the thermal stability of LAFs, CeO_2_, LAFs-CeO_2_ and SiO_2_-LAFs-CeO_2_ (Figure 2i). The LAFs exhibit a characteristic two-stage weight loss pattern, with an initial 9.81% mass loss below 100 °C attributed to moisture evaporation, followed by a major decomposition of the amyloid fibrils resulting in a total weight loss of 92.92% above 200 °C. This indicates that LAFs are stable below 200 °C. In contrast, pure CeO_2_ exhibits a gradual total weight loss of 8.1%, attributed to the removal of surface-adsorbed water and residues. The LAFs-CeO_2_ hybrid demonstrates a total weight loss of 63%. The significant reduction in decomposition mass loss compared to pure LAFs is directly attributed to the presence of the thermally stable CeO_2_ phase, which constitutes a substantial portion of the hybrid and remains as residue. Additionally, SiO_2_-LAFs-CeO_2_ composite showed the lowest total weight loss of 22%. This is primarily because the incorporated SiO_2_ significantly increases the proportion of non-degradable components in the system. Consequently, the relative content of the decomposable LAFs’ organic material is substantially reduced, leading to a much higher residual mass. TG results confirm the successful preparation of SiO_2_-doped amyloid fibril membranes loaded with CeO_2_.

### 3.2. Effect of the Dopant on the Permeability of LAFs

**Figure 3:**
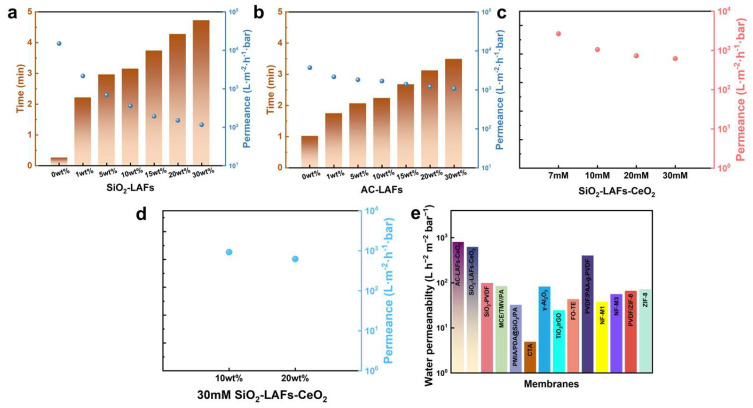
Water flux of LAFs. (**a**) The water flux of SiO_2_-doping LAFs. (**b**) The water flux of AC-doping LAFs. (**c**) The permeability of SiO_2_-LAFs-CeO_2_ with different amounts of CeO_2_. (**d**) The permeability of SiO_2_-LAFs-CeO_2_ with different amounts of LAFs-CeO_2_. (**e**) The comparison of the water flux of SiO_2_-LAFs-CeO_2_ and AC-LAFs-CeO_2_ with other membranes [36,37,38,39,40,41,42,43,44,45,46].

Prior to evaluating the fluoride removal performance, the permeability of the amyloid fibril-based hybrid membranes was analyzed. This was performed by measuring the time required for 10 mL of water to pass through membranes of identical thickness and dimensions. Results showed that the pure SiO_2_ membrane exhibited a high water flux of 38,000 L/(m^2^·h·bar) (Figure 3a). However, with the incorporation of LAFs, water flux gradually decreased, indicating reduced permeability and a corresponding increase in filtration time for the same volume of water. Similarly, the pure AC membrane initially demonstrated a high water flux of 41,000 L/(m^2^·h·bar), which also declined progressively upon the addition of LAFs (Figure 3b), leading to lower permeability and extended filtration duration. These findings suggest that pure LAF membranes have limited permeability, but doping with either SiO_2_ or AC effectively enhances their water permeation performance. A study on the effect of cerium oxide loading on the water permeability of SiO_2_-LAFs-CeO_2_ reveals that an increase in CeO_2_ content leads to a gradual decrease in water permeability (Figure 3c), indicating that higher CeO_2_ content is detrimental to the permeability of the hybrid membrane. Furthermore, as the content of LAFs-CeO_2_ in SiO_2_-LAFs-CeO_2_ increases, the water permeability also shows a declining trend (Figure 3d). The measured water flux values for SiO_2_-LAF-CeO_2_ and AC-LAF-CeO_2_ composite membranes reach up to 9000 and 8500 L/(m^2^·h·bar) (Figure 3c), respectively. These results indicate that both composite membranes exhibit significantly higher water permeability compared to many conventional membrane materials (Figure 3e and Table S1). This enhanced performance makes them highly suitable for efficient treatment of F-18 medical radioactive wastewater.

### 3.3. Fluoride Removal Performance

**Figure 4:**
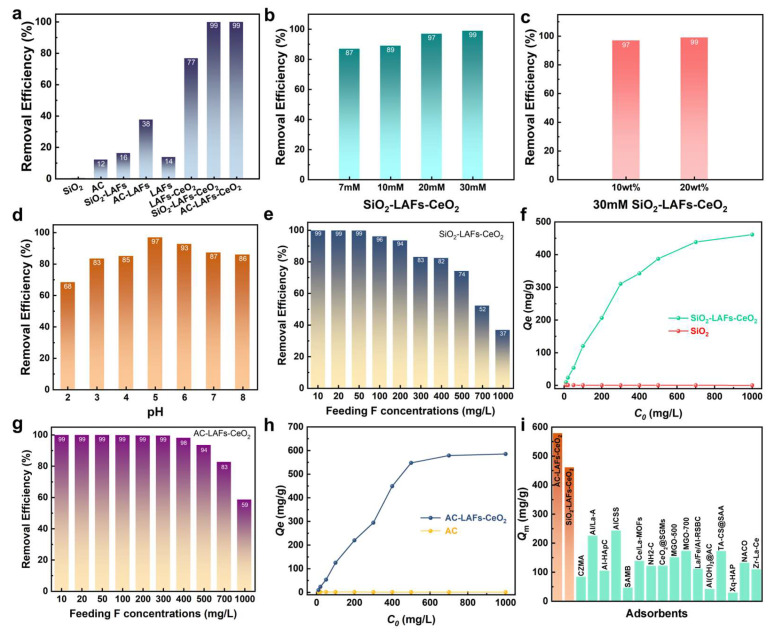
F^−^ removal performance. (**a**) The F^−^ removal performances of different membranes. (**b**) The influence of CeO_2_ content on the F^−^ removal performance of SiO_2_-LAFs-CeO_2_. (**c**) The influence of LAFs-CeO_2_ content on the F^−^ removal performance of SiO_2_-LAFs-CeO_2_. (**d**) The influence of solution pH on the F^−^ removal performance of SiO_2_-LAFs-CeO_2_. (**e**) The removal efficiency and (**f**) adsorption capacity of SiO_2_-LAFs-CeO_2_ in solutions with different F^−^ concentrations. (**g**) The removal efficiency and (**h**) adsorption capacity of AC-LAFs-CeO_2_ in solutions with different F^−^ concentrations. (**i**) The comparison of the F^−^ adsorption capacity between various membranes [47,48,49,50,51,52,53,54,55,56,57,58,59,60].

Based on the permeability analysis, we further investigated the fluoride removal performance of the various membrane materials. As illustrated in Figure 4a, the pure SiO_2_, pure AC and pure LAFs membranes all exhibit poor fluoride removal performance, with F^−^ removal rates below 14%. In contrast, the hybrid membranes SiO_2_-LAFs and AC-LAFs show a noticeable improvement, although their F^−^ removal rates remain below 38%. Notably, the LAFs-CeO_2_ membrane achieves a significantly higher F^−^ removal rate of 77%. A comparative analysis of the defluoridation performance among pure SiO_2_, pure AC, SiO_2_-LAFs, AC-LAFs, LAFs and LAFs-CeO_2_ reveals that SiO_2_, AC and LAFs exhibit weak adsorption toward F^−^, whereas CeO_2_ demonstrates a strong F^−^ adsorption capability. Moreover, SiO_2_-LAFs-CeO_2_ and AC-LAFs-CeO_2_ exhibit even greater fluoride removal efficiency than LAFs-CeO_2_, achieving F^−^ removal rates exceeding 99% under identical conditions. It is noteworthy that although an increase in the content of either CeO_2_ or LAFs-CeO_2_ leads to a decline in the water permeability of the SiO_2_-LAFs-CeO_2_ membrane, the F^−^ removal rate is enhanced (Figure 4b,c). These findings highlight that the SiO_2_-LAFs-CeO_2_ and AC-LAFs-CeO_2_ hybrid membranes are highly promising for fluorine-18 removal, an outcome attributed to the strong adsorption affinity of CeO_2_ toward fluoride and the enhanced porosity provided by the porous fillers.

The pH of fluorine-18-containing radioactive wastewater varies widely, making acid-base resistance an important consideration for the application of LAF membranes. Therefore, the effect of solution pH on the adsorption of fluoride ions by SiO_2_-LAFs-CeO_2_ membrane was systematically investigated. As shown in Figure 4d, SiO_2_-LAFs-CeO_2_ can effectively remove F^−^ through adsorption over a wide pH range (2–8), with removal rates consistently above 68% and reaching up to 97%. Due to the small dissociation constant (Ka) of hydrofluoric acid (HF), under acidic conditions (pH 2–4), some F^−^ combines with H^+^ to form HF, which hinders the interaction with the adsorbent, resulting in relatively lower removal rates of 68–85%. Under near-neutral conditions (pH 5–7), HF is completely dissociated into F^−^, leading to the highest adsorption efficiency, with removal rates reaching 87–97%. Under alkaline conditions, competitive adsorption between strong basic OH^−^ causes the removal rate to decrease to 86%. This result indicates that the SiO_2_-LAFs-CeO_2_ membrane can effectively treat neutral fluoride-containing wastewater, and also exhibits good performance for acidic and alkaline ones.

### 3.4. Saturated Adsorption Capacity

After confirming the effectiveness of SiO_2_-LAFs-CeO_2_ and AC-LAFs-CeO_2_ for F^−^ removal, their adsorption capacities are further investigated. By increasing the initial F^−^ concentration in the solution, the removal efficiency of F^−^ and the corresponding adsorption capacity of SiO_2_-LAFs-CeO_2_ and AC-LAFs-CeO_2_ are examined. As shown in Figure 4e, at initial concentrations of 10 to 50 mg/L, SiO_2_-LAFs-CeO_2_ effectively removes F^−^ from the solution with a removal efficiency exceeding 99%, indicating its potential for treating low-fluoride wastewater. As the F^−^ concentration increases, the removal efficiency gradually decreases. However, it remains above 93% at 200 mg/L, above 82% at 400 mg/L, and above 36% even at 1000 mg/L. Furthermore, the adsorption capacity of SiO_2_-LAFs-CeO_2_ increases with the initial F^−^ concentration (Figure 4f), reaching 461 mg/g at 1000 mg/L. On the other hand, AC-LAFs-CeO_2_ exhibits superior F^−^ adsorption performance. The removal efficiency remains above 98% at 400 mg/L, above 93% at 500 mg/L, and above 58% at 1000 mg/L (Figure 4g), demonstrating its potential for treating both low and high-concentration fluoride-containing wastewater. In addition, AC-LAFs-CeO_2_ shows a higher adsorption capacity, reaching 578.9 mg/g at an F^−^ concentration of 1000 mg/L (Figure 4h). This adsorption capacity is significantly higher than that of many existing membrane materials (Figure 4i and Table S2). It is noteworthy that the production cost of one ton of SiO_2_-LAFs-CeO_2_ is only 6949 USD, and its application in treating medical wastewater (with fluoride concentrations ranging from 7 to 2 mg/g) results in an overall cost of only 0.56 USD per ton (Table S3). These results further demonstrate the great potential of the cerium-loaded amyloid hybrid membrane in treating F-18-containing medical radioactive wastewater.

### 3.5. Interference Effect of Co-Existing Anions

**Figure 5:**
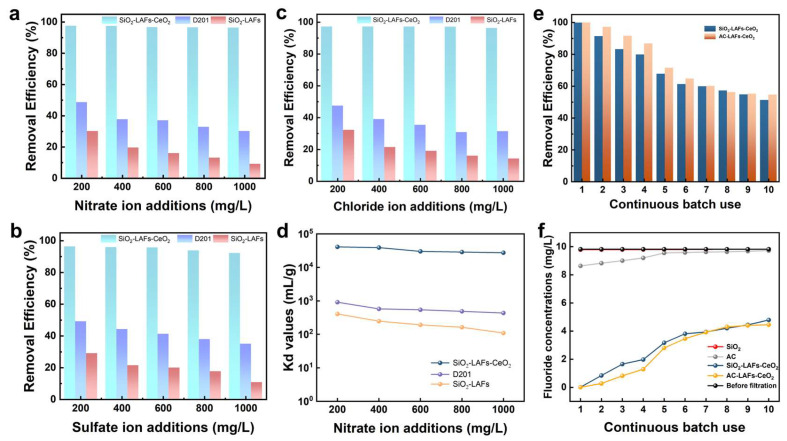
F^−^ removal performance. (**a**) Interference of nitrate on F^−^ adsorption. (**b**) Interference of sulfate on F^−^ adsorption. (**c**) Interference of chloride on F^−^ adsorption. (**d**) Distribution coefficient for F^−^ adsorption on various membranes. (**e**,**f**) The continuous batch use of SiO_2_-LAFs-CeO_2_ and AC-LAFs-CeO_2_.

In addition to fluoride ions (F^−^), medical wastewater also contains high concentrations of sulfate (SO_4_^2−^), nitrate (NO_3_^−^), and chloride (Cl^−^) ions, which may interfere with fluoride removal. Therefore, the influence of SO_4_^2−^, NO_3_^−^, and Cl^−^ on the F^−^ adsorption performance of SiO_2_-LAFs-CeO_2_ was investigated, with SiO_2_-LAFs and commercial resin D201 selected as comparative materials. As shown in Figure 5a, when exposed to high concentrations of NO_3_^−^ (200–1000 mg/L), the F^−^ removal efficiency of SiO_2_-LAFs-CeO_2_ remains unaffected, staying above 97%. This indicates a strong anti-interference capability against NO_3_^−^. In contrast, both SiO_2_-LAFs and commercial resin D201 show significant interference from NO_3_^−^, which intensifies with increasing concentration. At 200 mg/L NO_3_^−^, the F^−^ removal rates of SiO_2_-LAFs and D201 drop to 49.1% and 30.0%, respectively. When the NO_3_^−^ concentration reaches 1000 mg/L, the removal rates further decrease to 30.0% and 9.1%. Similarly, SO_4_^2−^ and Cl^−^ also considerably impair the F^−^ removal performance of SiO_2_-LAFs and D201. Notably, SiO_2_-LAFs-CeO_2_ exhibits strong resistance to both SO_4_^2−^ and Cl^−^ interference (Figure 5b,c). Even in the presence of high concentrations of SO_4_^2−^, its F^−^ removal efficiency remains above 97%, and in solutions containing chemically similar Cl^−^ ions, it still achieves over 95% removal. These results demonstrate that SiO_2_-LAFs-CeO_2_ possesses higher selectivity toward F^−^ compared to conventional adsorbents. To further quantify the selectivity difference among the three materials, the distribution coefficient (Kd) for F^−^ was calculated. As illustrated in Figure 5d, the K_d_ value of F^−^ adsorption onto SiO_2_-LAFs-CeO_2_ reaches the order of 10^4^, whereas the values for the other two materials remain below the order of 10^3^, confirming the superior selectivity of SiO_2_-LAFs-CeO_2_ for F^−^. This finding underscores the advantage of SiO_2_-LAFs-CeO_2_ in treating complex medical wastewater. For hospital wastewater containing fluorine-18, the radioactive effluent from imaging procedures is first collected in a shielded holding tank. The water is then pumped through the membrane module, where fluoride ions are adsorbed onto the cerium-loaded amyloid fibrils. Because the membrane exhibits high water permeability and high fluoride removal efficiency, a single pass can achieve more than 99% removal for low-concentration wastewater. For higher concentrations or larger volumes, multiple modules can be arranged in series or parallel. After the membrane reaches saturation, the module can be easily replaced and disposed of as radioactive solid waste.

### 3.6. Repeated Use of the Membranes

**Figure 6:**
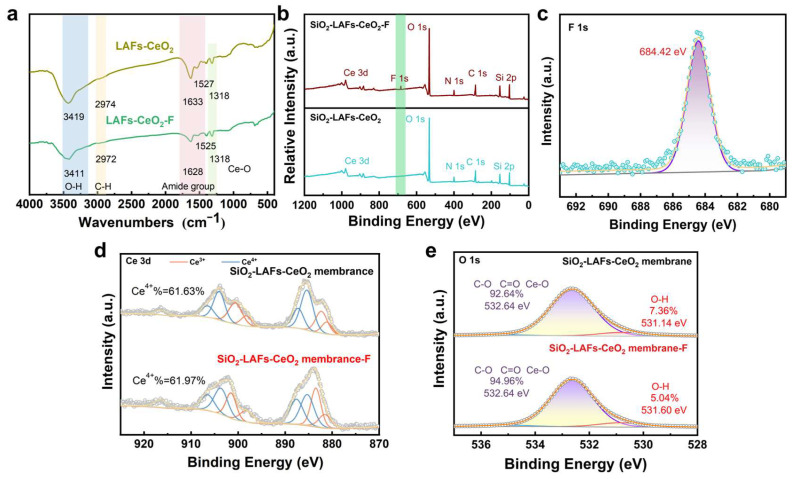
(**a**) The FT-IR spectra of LAFs-CeO_2_ before and after F^−^ adsorption. (**b**) The XPS full spectra of SiO_2_-LAFs-CeO_2_ before and after F^−^ adsorption. (**c**) The F 1s (**d**) Ce 3d and (**e**) O 1s XPS spectra of SiO_2_-LAFs-CeO_2_ before and after F^−^ adsorption.

The repeated use performance of an adsorbent is a crucial factor in determining its practical applicability. To assess this, the repeated use capacity of the SiO_2_-LAFs-CeO_2_ and AC-LAFs-CeO_2_ was evaluated over ten consecutive adsorption cycles using a 10 mg/L fluoride solution. After one adsorption cycle, the membranes are directly employed in the next batch of adsorption tests without regeneration. As shown in Figure 5e,f, the membrane maintained a fluoride removal efficiency of over 70% through the first five cycles. A gradual decline in efficiency was observed, with the removal rate reaching 51% by the tenth cycle. This attenuation is likely attributable to the progressive saturation of active adsorption sites, which gradually reduces the number of available sites for subsequent cycles. Despite the decline, the membrane retained significant adsorption capacity after ten uses. The minimal changes observed in the FT-IR and XRD spectra before and after use indicate that the composite structure remains largely intact (Figure 6a,b), confirming its potential for repeated application and underscoring the synergistic stability provided by the composite design.

### 3.7. Fluoride Removal Mechanism

XPS analysis was employed to elucidate the mechanism of fluoride adsorption onto the LAFS-CeO_2_ composite. As shown in Figure 6b, the survey scans confirmed the elemental composition before and after adsorption. Notably, the high-resolution F 1s spectrum of the spent adsorbent (Figure 6c) reveals a distinct new peak at a binding energy of 684.4 eV, which is entirely absent in the pristine material. This peak is characteristic of metal-fluoride (M-F) bonds, providing direct spectroscopic evidence for the successful chemisorption of F^−^ onto the SiO_2_-LAFS-CeO_2_ surface. A shift of the Ce 3d peaks toward higher binding energy is observed in the XPS spectrum after fluoride ion adsorption (Figure 6d). This shift indicates that the highly electronegative F^−^ substituted for the less electronegative O atoms in coordinating with Ce ions (Ce^3+^ and Ce^4+^). This substitution, due to the strong electron-withdrawing effect of F^−^, leads to an increase in the oxidation state of Ce and a positive shift in its binding energy. Further insight is gained from the deconvolution of the O 1s spectra (Figure 6b,c). The dominant component at approximately 531.0 eV in the pristine material, accounting for 7.36% of the oxygen signal, is attributed to metal-hydroxyl groups (M-OH). After F^−^ adsorption, this peak shifted slightly to 531.6 eV, and its relative abundance significantly decreased to 5.04%. The notable reduction in M-OH concentration, the increased oxidation state of Ce, and the emergence of a distinct F signal in the XPS spectrum provide strong support for the ion-exchange mechanism, wherein electronegative F^−^ displaces surface-bound OH^−^ [61].

## 4. Conclusions

This study successfully fabricated a novel hybrid membrane for fluoride removal by employing amyloid fibrils as a structural template for anchoring CeO_2_. The membrane was prepared through a straightforward process involving the oxidation–precipitation of cerium species onto the fibrils, followed by vacuum filtration, with the composition further tailored by incorporating porous silica or activated carbon dopants. The cerium-loaded amyloid fibril hybrid membrane featured a characteristic amyloid fibril structure. Benefiting from the doping with porous silica or activated carbon, it exhibited high water permeability, with a flux reaching up to 803.3 L/(m^2^·h·bar), significantly exceeding that of conventional membrane materials. Furthermore, the membrane efficiently adsorbs and removes F^−^ from both low and high-concentration fluoride-containing solutions, achieving a removal efficiency of up to 99% and a maximum adsorption capacity of 578.9 mg/g, which surpasses many other membrane materials. Additionally, the hybrid membrane demonstrates excellent resistance to ionic interference, enabling selective adsorption of F^−^ from solutions containing high concentrations of Cl^−^, NO^3−^, and SO_4_^2−^, with a distribution coefficient (Kd) as high as 4.1 × 10^4^ mL/g. The membrane retains 51% of its initial fluoride removal efficiency after 10 consecutive cycles without any intermediate regeneration, demonstrating potential for repeated use under continuous operation, while further regeneration strategies could improve long-term performance. Mechanistic studies indicate that the adsorption of F^−^ is primarily based on an ion exchange process between fluoride ions and the -OH groups on CeO_2_. This work presents a promising novel material with significant potential for the efficient treatment of medical radioactive wastewater containing fluorine-18.

## Data Availability

The original contributions presented in this study are included in this article. Further inquiries can be directed to the corresponding authors.

## Supplementary Materials

The following supporting information can be downloaded at: https://www.mdpi.com/article/10.3390/toxics14060490/s1, Table S1: Comparison of the water flux of SiO_2_-LAFs-CeO_2_ and AC-LAFs-CeO_2_ with other membranes; Table S2: Comparison of the F^−^ adsorption capacity between various membranes; Table S3: The economic cost evaluation for making membranes and use.
