# Supplementary material for: A Novel Cerium-Loaded Amyloid Hybrid Membrane for Advanced Removal of Fluorine-18 in Medical Wastewater
Source: Toxics. 2026 Jun 3;14(6):490. doi: 10.3390/toxics14060490 (PMC13306774; doi:10.3390/toxics14060490)
Supplement: Supplementary file 1 [file toxics-14-00490-s001.zip › toxics-4305758-supplementary.pdf]

Supporting Information

A novel cerium-loaded amyloid hybrid membrane for advanced removal of fluorine-18 in medical wastewater

Yue Xing 1, Fan Zhang1, 2 , Xu Zhang 1, *, Yuezhou Wei 1, 3, Chengtao Yue 1, Xiangbiao Yin 1, 4 *

1 School of Nuclear Science and Technology, University of South China, 28 Changsheng West Road, Hengyang 421001, PR China.

2 Division of Radiation Protection and Safety Control, Cyclotron and Radioisotope Center, Tohoku University, Sendai 980-8578, Japan.

3 School of Nuclear Science and Engineering, Shanghai Jiao Tong University, Shanghai 200240, China

4 Key Laboratory of Advanced Nuclear Energy Design and Safety, Ministry of Education, University of South China, Hengyang 421001, China

* Corresponding author: xuzhang2019@usc.edu.cn(X.Z.),
yinxb@usc.edu.cn(X.Y.),

Table S1. Comparison of the water flux of SiO_2-LAFs-CeO_2 and AC-LAFs-CeO_2 with other membranes

Membrane / Material	Water permeability L/(m^2·h·bar)	Reference
AC-LAFs-CeO_2	803.3	This work
SiO_2-LAFs-CeO_2	623	This work
SiO_2-PVDF	98.47	[36]
MCE/TMV/PA	84	[37]
PMIA/PDA@ SiO_2/PA	32.37	[38]
CTA	4.9	[39]
γ-Al_2O_3	82.07	[40]
TiO_2/rGO	24.5	[41]
FO-TE	43	[42]
PVDF/PAA-g-PVDF	400	[43]
NF-M1	37.7	[44]
NF-M3	55.6	[44]
PVDF/ZIF-8	66.19	[45]
ZIF-8	71.8	[46]

Table S2. Comparison of the F^- adsorption capacity between various membranes

Membrane / Material	Qe mg/g	Reference
AC-LAFs-CeO_2	578.9	This work
SiO_2-LAFs-CeO_2	461	This work
CZMA	84.24	[47]
Al/La-A	225	[48]
Al–HApC	105	[49]
AlCSS	242.8	[21]
SAMB	46.67	[50]
Ce-La-MOFs	138.64	[51]
NH2-C	121.12	[52]
CeO_2@SGMs	121.77	[53]
MGO-500	151.11	[54]
MGO-700	173.3	[54]
La/Fe/Al-RSBC	111	[55]
$Al(OH)_3$@AC	41.84	[56]
TA-CS@SAA	172.41	[57]
Xq-HAP	29.04	[58]
NACO	132	[59]
Zr-La-Ce	110	[60]

Table S3. The economic cost evaluation for making membranes and use

	β-lactoglobulin	SiO_2	AC	$CeCl_3 \cdot 7H_2O$	NaOH	HCl	H_2O	Other cost (1%)
**Mass(ton)**	0.2	0.8	0.8	0.003	0.05	0.01	5	
**Price(USD/ton)**	5428	5518.4	537.15	331960	406	147	0.3	
**Cost(USD)**	1085.6	4414.72	429.72	995.88	20.3	1.47	1.5	310
**Total cost(USD)**	6949 **USD/ton of adsorbent**							
**Treatment cost for F contaminated waters**								
**Electricity**	0.5 USD/ton of water							
**Treatment Capacity(ton water/ton adsorbent)**	116000							
**Treated cost**	0.56 USD/ton of water							

The price was obtained from www.alibaba.com
